# Approximations for the inverse cumulative distribution function of the gamma distribution used in wireless communication

**DOI:** 10.1016/j.heliyon.2020.e05523

**Published:** 2020-11-18

**Authors:** Hilary Okagbue, Muminu O. Adamu, Timothy A. Anake

**Affiliations:** a Department of Mathematics, Covenant University, Ota, Nigeria; b Department of Mathematics, University of Lagos, Akoka, Lagos, Nigeria

**Keywords:** Electrical engineering, Safety engineering, Statistics, Risk analysis, Quantile function, Inverse cumulative distribution function, Gamma distribution, Ordinary differential equation, Shape parameter, Wireless communication, Kullback-Leibler divergence

## Abstract

The use of quantile functions of probability distributions whose cumulative distribution is intractable is often limited in Monte Carlo simulation, modeling, and random number generation. Gamma distribution is one of such distributions, and that has placed limitations on the use of gamma distribution in modeling fading channels and systems described by the gamma distribution. This is due to the inability to find a suitable closed-form expression for the inverse cumulative distribution function, commonly known as the quantile function (QF). This paper adopted the Quantile mechanics approach to transform the probability density function of the gamma distribution to second-order nonlinear ordinary differential equations (ODEs) whose solution leads to quantile approximation. Closed-form expressions, although complex of the QF, were obtained from the solution of the ODEs for degrees of freedom from one to five. The cases where the degree of freedom is not an integer were obtained, which yielded values closed to the R software values via Monte Carlo simulation. This paper provides an alternative for simulating gamma random variables when the degree of freedom is not an integer. The results obtained are fast, computationally efficient and compare favorably with the machine (R software) values using absolute error and Kullback–Leibler divergence as performance metrics.

## 1 Introduction

The gamma distribution, in its simplest form, is characterized by two positive parameters known as the degrees of freedom or the shape parameter, k, and the rate or shape parameter. The distribution is directly related to the normal, Chi, Chi-square, beta, Nakagami, Erlang and other probability distributions. The distribution has been used for modeling in financial modeling and engineering, epidemiology, biostatistics and biometrics, computational biology and neuroscience, soft computing, meteorology, geophysics, engineering, and so on.

Gamma Distribution has been applied to the domain of wireless communication in diverse ways. A review of recent applications revealed that the use could be classified into six major areas.

• Modeling fading channels, shadowing effects [1, 2, 3, 4, 5, 6, 7, 8, 9, 10, 11] and attenuation in wireless networks [12];
• Other forms of modeling such as: binary error modeling [13], beamforming [14], spatial deployment modeling [15], delay [16], source localization [17], line of sight interference power [18], atmospheric turbulence [19, 20] and color texture characterization [21].
• Modeling by the direct use of gamma distribution fit via parameter estimation [22, 23, 24].
• Approximating a phenomenon, for example, signal and interference powers [25, 26], co-channel interference [27], rendezvous time [28], simulation results in fading wireless channel conditions [29], action duration and inter-arrival [30] and resource requirements for traffic characteristics [31], and
• Derivation of closed forms of models that follows gamma distribution. The closed forms are useful in modelling wireless network systems and other related models as seen in beamforming [32], bit error rate [33, 34] and average bit error rate [35].

## 2 Gamma distribution

Gamma distribution is a two-parameter family of continuous probability distributions. The basics of gamma distribution are presented in this section. Generally, the support of the distribution and the range of the parameters are given as; x∈(0, ∞), k>0, λ>0.

The probability density function f(x) and the cumulative distribution function F(x) are given as:

(1) $$f\left(x\right)=\frac{\lambda^{k}}{\Gamma \left(k\right)}x^{k-1}e^{-\lambda x}$$

Where Γ(k) is the gamma function.

(2) $$F\left(x\right)=\int_{0}^{x}f\left(w,k,\lambda \right)dw=\frac{\gamma \left(k,\lambda x\right)}{\Gamma \left(k\right)}$$

Where γ(k,λx) is the lower incomplete gamma function. If the shape parameter k is strictly a positive integer, the distribution reduces to the Erlang distribution. The mean, mode, variance, skewness, excess kurtosis (EK), entropy, moment generating function (MGF), characteristic function (CF) and logarithmic expression (LE) of the distribution are presented in Table 1.

**Table 1:** Summary of mathematical function and expressions for the gamma distribution.

Function	Mathematical Expression
Mean	$\frac{k}{\lambda }$
Mode	$\frac{k-1}{\lambda }$ for k≥1
Variance	$\frac{k}{\lambda^{2}}$
Skewness	$\frac{2}{\sqrt{k}}$
EK	$\frac{6}{k}$
Entropy	k−lnλ+lnΓ(k)+(1−k)φ(k)
MGF	${\left(1-\frac{t}{\lambda }\right)}^{-k}$ for t<λ
CF	${\left(1-\frac{it}{\lambda }\right)}^{-k}$
LE	φ(k)−φ(λ)

φ = Digamma function.

It can be seen from Table 1 that there is no simple closed-form expression for the median and that explains its exclusion there. This is due to the intractable nature of the CDF as it cannot be easily transformed to obtain the quantile function (QF), of which the median is a particular case. This is not limited to the median, but other quartiles such as the decile, quintile, lower quartile, and upper quartile. The absence of the closed-form expression for both the CDF and QF guarantee that the closed-form expressions for the inverse survival function (ISF), survival function (SF), odds function (OF), hazard function (HF) and reversed hazard function (RHF) are disappointedly unavailable. The alternative, followed by many researchers, is the use of approximations, which can be in the form of a series, functional, proxy, or the use of numerical algorithms and, recently, the use of numerical optimization methods [36]. Ultimately, the relevance of the QF is mostly due to its utilization in simulation and modeling, random number generation and copulas [37, 38, 39].

This paper applies the Quantile Mechanics (QM) approach that transforms PDF of complex distributions into second-order nonlinear ordinary differential equations whose solutions, when modified, can provide series, closed-form, or closed-form equations of the quantile function of the studied distribution [40]. One of the major advantages of QM is that it has proven to be very efficient in quantile approximation of complex and distributions with shape parameters [41, 42]. The solutions are given per value of the shape parameter because distributions with shape parameters are often difficult in obtaining their approximation [42]. The existing approximations are often slow [43, 44], plagued with slow convergence [45, 46, 47] and cumbersome in dealing with the extreme tails of complex distributions [48, 49, 50, 51, 52]. On the other hand, the second-order nonlinear ODE generated using QM is often complex. The solutions may be very difficult to obtain and hence, limiting the acceptability of the method among researchers.

The R software uses the algorithm [53] based on the relationship between the gamma and Chi-square distributions. Regrettably, no closed-form is available except when the shape parameter equals one. The absence of the closed-form limits its application. Also, the intractability of the distribution limits its compounding with other distribution, which often improves its use in modeling real-life events.

QM was used in this paper to obtain the closed-form of the QF of the gamma distribution. Degrees of freedom up to five and improper fractions were considered. The complexity of the distribution necessitated that the QF obtained for each degree of freedom is different from others. A comparison with the machine values was done to evaluate the efficiency and the performance of the quantile models obtained. Recently, authors [54] applied the same methodology proposed in this paper to obtained the near exact quantile values for the Erlang distribution. Unfortunately, the results cannot be applied to the gamma distribution since Erlang is a subset of the gamma distributions. Erlang distribution considers only the cases of gamma, where the shape parameter is a positive integer. The implication is that the result of [54] cannot be applied when the shape parameter is not a positive integer.

## 3 Model formulation

Quantile Mechanics approach is applied to the PDF of the gamma distribution, thereby transforming Eq. (1) into;

(3) $$\frac{dQ}{dp}=\frac{\Gamma \left(k\right)}{\lambda^{k}}Q^{1-k}\left(p\right){\mathrm{e}}^{\lambda Q\left(p\right)}$$

The result is similar to [40].

Differentiate to obtain;

(4) $$\frac{d^{2}Q}{dp^{2}}=\frac{\Gamma \left(k\right)}{\lambda^{k}}\left[Q^{1-k}\left(p\right)\lambda {\mathrm{e}}^{\lambda Q\left(p\right)}\frac{dQ}{dp}+\left(1-k\right)Q^{-k}\left(p\right){\mathrm{e}}^{\lambda Q\left(p\right)}\frac{dQ}{dp}\right].$$

(5) $$\frac{d^{2}Q}{dp^{2}}=\frac{\Gamma \left(k\right)}{\lambda^{k}}\left[Q^{1-k}\left(p\right){\mathrm{e}}^{\lambda Q\left(p\right)}\lambda +\left(1-k\right)\frac{Q^{1-k}}{Q^{1-k}}Q^{-k}\left(p\right){\mathrm{e}}^{\lambda Q\left(p\right)}\right]\frac{dQ}{dp}$$

Substitute Eq. (3) into Eq. (5) to obtain;

(6) $$\frac{d^{2}Q}{dp^{2}}=\left[\lambda +\left(1-k\right)\frac{Q^{-k}}{Q^{1-k}}\right]{\left(\frac{dQ}{dp}\right)}^{2}$$

(7) $$\frac{d^{2}Q}{dp^{2}}=\left[\lambda +\frac{1-k}{Q}\right]{\left(\frac{dQ}{dp}\right)}^{2}$$

Eq. (7) can be referred to the gamma distribution differential equation (GDDE) and the solution gives the required quantile function and can be solved using the assumed initial value conditions $Q\left(0\right)=0,\;Q^{\prime }\left(0\right)=1$. Details on the review of the method and similar methodologies can be found in [55, 56, 57, 58, 59].

## 4 Results

Results are obtained for degrees of freedom from one to five. The steps outlined in this paper can be followed to obtain the closed-form expression of the gamma distribution for any given shape parameter or degrees of freedom.

### 4.1 Shape parameter equals one

Gamma distribution has closed form expression for the CDF and QF at k = 1. This is because at k = 1, gamma distribution reduces to the exponential. The solution of GDDE at k = 1, is;

(8) $$Q\left(p\right)=\frac{1}{\lambda }\ln\left(\frac{1}{1-p}\right)$$

Eq. (8) is the QF of the exponential distribution which can easily be inverted to obtain the CDF as $F\left(x\right)=1-e^{-\lambda x}$.

The distribution has no closed form expressions for both the CDF and QF except at k = 1.

### 4.2 Shape parameter equals two

This is the particular case of Eq. (7) when k = 2 and λ=1. This implies that Eq. (7) reduces to;

(9) $$\frac{d^{2}Q}{dp^{2}}=\left[1-\frac{1}{Q}\right]{\left(\frac{dQ}{dp}\right)}^{2}$$

The solution of Eq. (9) was obtained explicitly without the use of the initial values. The solution is presented as;

(10) $$Q(p)=W\left(\frac{c_{1}(p+c_{2})}{e}\right)-1$$

Where W is the Lambert function used mostly in complex number analysis, e is the exponential function and c_1_ and c_2_ are the initial values. The inability to use a specific value of the initial values is because of the absence of closed-form and the problematic nature of quantiles at extreme tails and as such, boundary values are not feasible. The tolerable alternative is to assign values to the initial value conditions. This is due to infinitely many solutions that are available. Secondly, because of the presence of the Lambert function, near zero values of the initial values are used to annul the effect of the Lambert function. Mathematically, near zero values are almost unaffected by Lambert function transformation.

Numerical values were therefore assigned to the initial values; $c_{1}=-0.0001$, $c_{2}=1.0001$ and the exponential function is commonly known to be 2.718281828. Negative value was assigned to c_1_ to shield Eq. (10) from having negative outcomes. The values are incorporated into Eq. (10) to obtain;

(11) Q(p)+1=W(0.000036788(p+1.0001))

The complexity of Eq. (10) means that the quartiles has to be obtained individually and fitting is done with the R software values in order to obtain the closed form of the quantile function for the degrees of freedom equal to two.

The computations are given as follows;

1). When p = 0.1 and the R software quantile values at 0.1 is 0.531811608.

Substitute into Eq. (10) to obtain;

(12) 0.531811608+1=W(0.000036788(1.1001))

(13) 1.531811608=W(0.00004047)

The Lambert function is unaffected by small values.

(14) 1.531811608=A(0.00004047)

Because of the availability of infinite solutions, the difference between the L.H.S. and R.H.S of Eq. (14) is when the R.H.S. is multiply with A = 37850.09848. Finally, the quantile function of gamma distribution at p = 0.10 (decile) and k = 2 is given by;

(15) Q(p)=1.392429423(p+1.0001)−1

Similarly, other percentage points or quartiles were obtained using the approach and can be seen in Table 2. Also, presented are the results for the extreme tails of the distribution shown in Table 3.

**Table 2:** Closed form of the gamma function for degrees of freedom equals two.

p	Q(p) (closed form)
0.1	1.392429423(p+1.0001)−1
0.2	1.520196908(p+1.0001)−1
0.3	1.613221453(p+1.0001)−1
0.4	1.697322578(p+1.0001)−1
0.5	1.78544563(p+1.0001)−1
0.6	1.888827726(p+1.0001)−1
0.7	2.022949522(p+1.0001)−1
0.8	2.218936919(p+1.0001)−1
0.9	2.573401489(p+1.0001)−1

**Table 3:** Closed form for the extreme tails of the gamma function for degrees of freedom equals two.

p	Q(p) (closed form)	p	Q(p) (closed form)
0.01	1.13707033(p+1.0001)−1	0.91	2.629033896(p+1.0001)−1
0.02	1.190764724(p+1.0001)−1	0.92	2.69166494(p+1.0001)−1
0.03	1.230489115(p+1.0001)−1	0.93	2.763180207(p+1.0001)−1
0.04	1.262929122(p+1.0001)−1	0.94	2.846339974(p+1.0001)−1
0.05	1.290697563(p+1.0001)−1	0.95	2.9454205(p+1.0001)−1
0.06	1.315146195(p+1.0001)−1	0.96	3.067578003(p+1.0001)−1
0.07	1.337085024(p+1.0001)−1	0.97	3.226206358(p+1.0001)−1
0.08	1.357046386(p+1.0001)−1	0.98	3.451301299(p+1.0001)−1
0.09	1.37540323(p+1.0001)−1	0.99	3.838175(p+1.0001)−1

### 4.3 Shape parameter equals three

This is the particular case of Eq. (7) when k = 3 and λ=1. This implies that Eq. (7) reduces to;

(16) $$\frac{d^{2}Q}{dp^{2}}=\left[1-\frac{2}{Q}\right]{\left(\frac{dQ}{dp}\right)}^{2}$$

The solution of Eq. (16) was obtained explicitly without the use of the initial values. The solution is presented as a complex closed form equation;

(17) $$c_{1}+p=\frac{e^{-Q\left(p\right)}\left(Q^{2}\left(p\right)+2Q\left(p\right)+2\right)}{c_{1}}$$

(18) $$c_{1}^{2}+c_{1}p=e^{-Q\left(p\right)}\left(Q^{2}\left(p\right)+2Q\left(p\right)+2\right)$$

Fitting is done on Eq. (18) using the R software values of the quantile function. Hence, the right-hand-side of Eq. (18) for different values of p are given in Table 4.

**Table 4:** The values of $R\left(p\right)=e^{-Q\left(p\right)}\left(Q^{2}\left(p\right)+2Q\left(p\right)+2\right)$ for different quartiles.

p	R(p)	p	R(p)	p	R(p)
0.01	1.98	0.1	1.80	0.91	0.18
0.02	1.96	0.2	1.60	0.92	0.16
0.03	1.94	0.3	1.40	0.93	0.14
0.04	1.92	0.4	1.20	0.94	0.12
0.05	1.90	0.5	1.00	0.95	0.10
0.06	1.88	0.6	0.80	0.96	0.08
0.07	1.86	0.7	0.60	0.97	0.06
0.08	1.84	0.8	0.40	0.98	0.04
0.09	1.82	0.9	0.20	0.99	0.02

It can be seen that the pattern from Table 4 connotes that R(p) decreases as p approaches 1 and also exist on a bounded interval given as;

(19) 0<R(p)<2

The contents of Table 4 are used to solve for $c_{1}$ of Eq. (18). Two examples are provided.

When p = 0.1, using Table 4, Eq. (18) becomes;

$c_{1}^{2}+c_{1}p=1.8$, $c_{1}^{2}+0.1c_{1}=1.8$, $c_{1}^{2}+0.1c_{1}-1.8=0$. Solving for $c_{1}$ gives;

(20) $$c_{1}=\frac{-0.1\pm 2.685144316}{2}=1.292572158$$

When p = 0.2, using Table 4, Eq. (18) becomes;

$c_{1}^{2}+c_{1}p=1.6$, $c_{1}^{2}+0.2c_{1}=1.6$, $c_{1}^{2}+0.2c_{1}-1.6=0$.

Solving for $c_{1}$ gives;

(21) $$c_{1}=\frac{-0.2\pm 2.537715508}{2}=1.168857754$$

Inserting the value of $c_{1}$ makes the R.H.S and the L.H.S of Eq. (18) to be given for p=0.1 and p=0.2 respectively. The process is repeated for other quartiles and are summarized in Table 5. This becomes the closed form expressions for the QF of the gamma distribution for this case.

**Table 5:** Values of $c_{1}$ for the QF of gamma distribution at degrees of freedom equals three.

p	$c_{1}$	p	$c_{1}$	p	$c_{1}$
0.01	1.402133611	0.1	1.292572158	0.91	0.167113333
0.02	1.390035714	0.2	1.168857754	0.92	0.149590026
0.03	1.377919596	0.3	1.042686044	0.93	0.131845876
0.04	1.365784976	0.4	0.913552872	0.94	0.113866423
0.05	1.353631568	0.5	0.780776406	0.95	0.09563561
0.06	1.341459077	0.6	0.643398113	0.96	0.077135531
0.07	1.329267203	0.7	0.50	0.97	0.058346114
0.08	1.317055636	0.8	0.348331476	0.98	0.039244744
0.09	1.304824063	0.9	0.184428877	0.99	0.019805788

It can be seen that the pattern from Table 5 connotes that $c_{1}$ decreases as p approaches 1 and also exist on a bounded interval given as;

(22) $$0<c_{1}<2$$

### 4.4 Shape parameter equals four

This is the particular case of Eq. (7) when k = 4 and λ=1. This implies that Eq. (7) reduces to;

(23) $$\frac{d^{2}Q}{dp^{2}}=\left[1-\frac{3}{Q}\right]{\left(\frac{dQ}{dp}\right)}^{2}$$

The solution of Eq. (23) was obtained explicitly without the use of the initial values. The solution is presented as a complex closed form equation;

(24) $$c_{1}+p=\frac{e^{-Q\left(p\right)}\left(Q^{3}\left(p\right)+3Q^{2}\left(p\right)+6Q\left(p\right)+6\right)}{c_{1}}$$

(25) $$c_{1}^{2}+c_{1}p=e^{-Q\left(p\right)}\left(Q^{3}\left(p\right)+3Q^{2}\left(p\right)+6Q\left(p\right)+6\right)$$

Fitting is done on Eq. (25) using the R software values of the quantile function. Hence, the right-hand-side of Eq. (25) for different values of p are given in Table 6.

**Table 6:** The values of $R\left(p\right)=e^{-Q\left(p\right)}\left(Q^{3}\left(p\right)+3Q^{2}\left(p\right)+6Q\left(p\right)+6\right)$ for different quartiles.

p	R(p)	p	R(p)	p	R(p)
0.01	5.94	0.1	5.4	0.91	0.54
0.02	5.88	0.2	4.8	0.92	0.48
0.03	5.82	0.3	4.2	0.93	0.42
0.04	5.76	0.4	3.6	0.94	0.36
0.05	5.70	0.5	3.0	0.95	0.30
0.06	5.64	0.6	2.4	0.96	0.24
0.07	5.58	0.7	1.8	0.97	0.18
0.08	5.52	0.8	1.2	0.98	0.12
0.09	5.46	0.9	0.6	0.99	0.06

It can be seen that the pattern from Table 6 connotes that R(p) decreases as p approaches 1 and also exist on a bounded interval given as;

(26) 0<R(p)<6

The contents of Table 6 are used to solve for $c_{1}$ of Eq. (25). Two examples are provided.

When p = 0.1, using Table 6, Eq. (25) becomes;

$c_{1}^{2}+c_{1}p=5.4$, $c_{1}^{2}+0.1c_{1}=5.4$, $c_{1}^{2}+0.1c_{1}-5.4=0$. Solving for $c_{1}$ gives;

(27) $$c_{1}=\frac{-0.1\pm 4.64865572}{2}=2.27432786$$

When p = 0.2, using Table 6, Eq. (25) becomes;

$c_{1}^{2}+c_{1}p=4.8$, $c_{1}^{2}+0.2c_{1}=4.8$, $c_{1}^{2}+0.2c_{1}-4.8=0$. Solving for $c_{1}$ gives;

(28) $$c_{1}=\frac{-0.2\pm 4.38634244}{2}=2.09317122$$

Inserting the value of $c_{1}$ makes the R.H.S and the L.H.S of Eq. (25) to be given for p=0.1 and p=0.2 respectively. The process is repeated for other quartiles and are summarized in Table 7. This becomes the closed form expressions for the QF of the gamma distribution for this case.

**Table 7:** Values of $c_{1}$ for the QF of gamma distribution at degrees of freedom equals four.

p	$c_{1}$	p	$c_{1}$	p	$c_{1}$
0.01	2.43221665	0.1	2.27432786	0.91	0.40930608
0.02	2.41489175	0.2	2.09317122	0.92	0.371624915
0.03	2.397514249	0.3	1.904872259	0.93	0.332637135
0.04	2.380083332	0.4	1.707878403	0.94	0.29216796
0.05	2.362598166	0.5	1.50	0.95	0.25
0.06	2.345057894	0.6	1.277973384	0.96	0.205857128
0.07	2.32746164	0.7	1.036542462	0.97	0.159379546
0.08	2.309808503	0.8	0.766190379	0.98	0.110083327
0.09	2.292097559	0.9	0.445823643	0.99	0.057290684

It can be seen that the pattern from Table 7 connotes that $c_{1}$ decreases as p approaches 1 and also exist on a bounded interval given as;

(29) $$0<c_{1}<3$$

### 4.5 Shape parameter equals five

This is the particular case of Eq. (7) when k = 5 and λ=1. This implies that Eq. (7) reduces to;

(30) $$\frac{d^{2}Q}{dp^{2}}=\left[1-\frac{4}{Q}\right]{\left(\frac{dQ}{dp}\right)}^{2}$$

The solution of Eq. (30) was obtained explicitly without the use of the initial values. The solution is presented as a complex closed form equation;

(31) $$c_{1}+p=\frac{e^{-Q\left(p\right)}\left(Q^{4}\left(p\right)+4Q^{3}\left(p\right)+12Q^{2}\left(p\right)+24Q\left(p\right)+24\right)}{c_{1}}$$

(32) $$c_{1}^{2}+c_{1}p=e^{-Q\left(p\right)}\left(Q^{4}\left(p\right)+4Q^{3}\left(p\right)+12Q^{2}\left(p\right)+24Q\left(p\right)+24\right)$$

Fitting is done on Eq. (32) using the R software values of the quantile function. Hence, the right-hand-side of Eq. (32) $R\left(p\right)=e^{-Q\left(p\right)}\left(Q^{4}\left(p\right)+4Q^{3}\left(p\right)+12Q^{2}\left(p\right)+24Q\left(p\right)+24\right)$ for different values of p are given in Table 8.

**Table 8:** The values of $R\left(p\right)=e^{-Q\left(p\right)}\left(Q^{4}\left(p\right)+4Q^{3}\left(p\right)+12Q^{2}\left(p\right)+24Q\left(p\right)+24\right)$ for different quartiles.

p	R(p)	p	R(p)	p	R(p)
0.01	23.76	0.1	21.6	0.91	2.16
0.02	23.52	0.2	19.2	0.92	1.92
0.03	23.28	0.3	16.8	0.93	1.68
0.04	23.04	0.4	14.4	0.94	1.44
0.05	22.80	0.5	12.0	0.95	1.20
0.06	22.56	0.6	9.6	0.96	0.96
0.07	22.32	0.7	7.2	0.97	0.72
0.08	22.08	0.8	4.8	0.98	0.48
0.09	21.84	0.9	2.4	0.99	0.24

It can be seen that the pattern from Table 8 connotes that R(p) decreases as p approaches 1 and also exist on a bounded interval given as;

(33) 0<R(p)<24

The contents of Table 8 are used to solve for $c_{1}$ of Eq. (32). Two examples are provided.

When p = 0.1, using Table 8, Eq. (32) becomes;

$c_{1}^{2}+c_{1}p=21.6$, $c_{1}^{2}+0.1c_{1}=21.6$, $c_{1}^{2}+0.1c_{1}-21.6=0$. Solving for $c_{1}$ gives;

(34) $$c_{1}=\frac{-0.1\pm 9.29569793}{2}=4.597848965$$

When p = 0.2, using Table 6, Eq. (32) becomes;

$c_{1}^{2}+c_{1}p=19.2$, $c_{1}^{2}+0.2c_{1}=19.2$, $c_{1}^{2}+0.2c_{1}-19.2=0$. Solving for $c_{1}$ gives;

(35) $$c_{1}=\frac{-0.2\pm 8.7658428}{2}=4.2829214$$

Inserting the value of $c_{1}$ makes the R.H.S and the L.H.S of Eq. (32) to be given for p=0.1 and p=0.2 respectively. The process is repeated for other quartiles and are summarized in Table 9. This becomes the closed form expressions for the QF of the gamma distribution for this case.

**Table 9:** Values of $c_{1}$ for the QF of gamma distribution at degrees of freedom equals five.

p	$c_{1}$	p	$c_{1}$	p	$c_{1}$
0.01	4.869425607	0.1	4.597848965	0.91	1.083513893
0.02	4.839752571	0.2	4.2829214	0.92	1.00
0.03	4.809958549	0.3	3.951524107	0.93	0.912034857
0.04	4.780041666	0.4	3.60	0.94	0.818759093
0.05	4.75	0.5	3.223110997	0.95	0.718995393
0.06	4.719831576	0.6	2.812876483	0.96	0.611054535
0.07	4.689534369	0.7	2.356011826	0.97	0.492356127
0.08	4.659106298	0.8	1.827105745	0.98	0.35858706
0.09	4.628545228	0.9	1.16322658	0.99	0.201437362

It can be seen that the pattern from Table 9 connotes that $c_{1}$ decreases as p approaches 1 and also exist on a bounded interval given as;

(36) $$0<c_{1}<6$$

### 4.6 Cases when the shape parameter is not an integer

#### 4.6.1 When K = 1.5

This is the particular case of Eq. (7) when k = 1.5 and λ=1. This implies that Eq. (7) reduces to;

(37) $$\frac{d^{2}Q}{dp^{2}}=\left[1-\frac{1}{2Q}\right]{\left(\frac{dQ}{dp}\right)}^{2}$$

The solution of Eq. (37) was obtained explicitly without the use of the initial values. The solution is presented as a complex closed form equation;

(38) $$c_{1}+p=\frac{\frac{1}{2}\sqrt{p}\;erf\left(\sqrt{Q(p)}\right)-e^{-Q(p)}\sqrt{Q(p)}}{c_{2}}$$

(39) $$c_{1}c_{2}+c_{2}p=\frac{1}{2}\sqrt{p}\;erf\left(\sqrt{Q(p)}\right)-e^{-Q(p)}\sqrt{Q(p)}$$

Fitting is done on Eq. (39) using the R software values of the quantile function. Hence, the right-hand-side of Eq. (39) $R(p)=\frac{1}{2}\sqrt{p}\;erf\left(\sqrt{Q(p)}\right)-e^{-Q(p)}\sqrt{Q(p)}$ for different values of p are given in Table 10.

**Table 10:** The values of $R(p)=\frac{1}{2}\sqrt{p}\;erf\left(\sqrt{Q(p)}\right)-e^{-Q(p)}\sqrt{Q(p)}$ for different quartiles.

p	R(p)	p	R(p)	p	R(p)
0.01	0.8520253	0.1	1.273077022	0.91	-0.778481135
0.02	1.014943147	0.2	1.143783386	0.92	-0.803514652
0.03	1.108230433	0.3	0.905747558	0.93	-0.828423737
0.04	1.168753556	0.4	0.630853727	0.94	-0.853211384
0.05	1.209707567	0.5	0.344020359	0.95	-0.877881961
0.06	1.237501819	0.6	0.056975554	0.96	-0.902441945
0.07	1.255785337	0.7	-0.22385365	0.97	-0.926901348
0.08	1.266853167	0.8	-0.494671174	0.98	-0.951276969
0.09	1.272251697	0.9	-0.753321149	0.99	-0.97560221

It can be seen from Table 10 that the values of R(p) decreases to zero for low values of p and approximates to -1 as p tends towards 1. Hence; $\lim_{R\left(p\right)\to 0}=0$ and $\lim_{R\left(p\right)\to 1}=-1$

Initial values are assumed for $c_{2}=1$ and $c_{1}$ is obtained for different values of the quartiles that will make

(40) $$c_{1}+p=R(p)=\frac{1}{2}\sqrt{p}\;erf\left(\sqrt{Q(p)}\right)-e^{-Q(p)}\sqrt{Q(p)}$$

The values of $c_{1}$ for different quartiles are presented in Table 11.

**Table 11:** Values of $c_{1}$ for different quartiles for k = 1.5

p	$c_{1}$	p	$c_{1}$	p	$c_{1}$
0.01	-0.1479747	0.1	0.273077022	0.91	-1.778481135
0.02	0.014943147	0.2	0.143783386	0.92	-1.803514652
0.03	0.108230433	0.3	-0.094252442	0.93	-1.828423737
0.04	0.168753556	0.4	-0.369146273	0.94	-1.853211384
0.05	0.209707567	0.5	-0.655979641	0.95	-1.877881961
0.06	0.237501819	0.6	-0.943024446	0.96	-1.902441945
0.07	0.255785337	0.7	-1.22385365	0.97	-1.926901348
0.08	0.266853167	0.8	-1.494671174	0.98	-1.951276969
0.09	0.272251697	0.9	-1.753321149	0.99	-1.97560221

It can be seen from Table 11 that the values of $c_{1}$ decreases to -1 for low values of p and approximates to -2 as p tends towards 1. Hence; $\lim_{c_{1}\to 0}=-1$ and $\lim_{c_{1}\to 1}=-2$

The complexity of Eq. (40) implies that Q(p) cannot be easily simplified. Hence simulation can be done using;

(41) Q(p)=A(p)R(p)

Where A(p) is computed using the R software values. The values of A(p) are presented in Table 12.

**Table 12:** Values of A(p) for different quartiles for k = 1.5

p	A(p)	p	A(p)	p	A(p)
0.01	0.067387554	0.1	0.229512576	0.91	-4.169309587
0.02	0.091055257	0.2	0.439407507	0.92	-4.205705899
0.03	0.110581113	0.3	0.785899024	0.93	-4.261293982
0.04	0.128406633	0.4	1.481459427	0.94	-4.340589088
0.05	0.145426187	0.5	3.438712013	0.95	-4.450899011
0.06	0.162090007	0.6	25.85465069	0.96	-4.604823034
0.07	0.178668554	0.7	-8.185863347	0.97	-4.826450799
0.08	0.195345275	0.8	-4.691629426	0.98	-5.170633594
0.09	0.212256739	0.9	-4.149218853	0.99	-5.814289174

It can be seen from Table 12 that the values of A(p) decreases to 0 for low values of p and approximates to -6 as p tends towards 1. Hence; $\lim_{A\left(p\right)\to 0}=Q{\left(p\right)}^{2}$ and $\lim_{A\left(p\right)\to 1}=-Q\left(p\right)$ (This was the result as $p=10^{-56}$ and p≈1.

#### 4.6.2 Simulation

**Figure 1:**
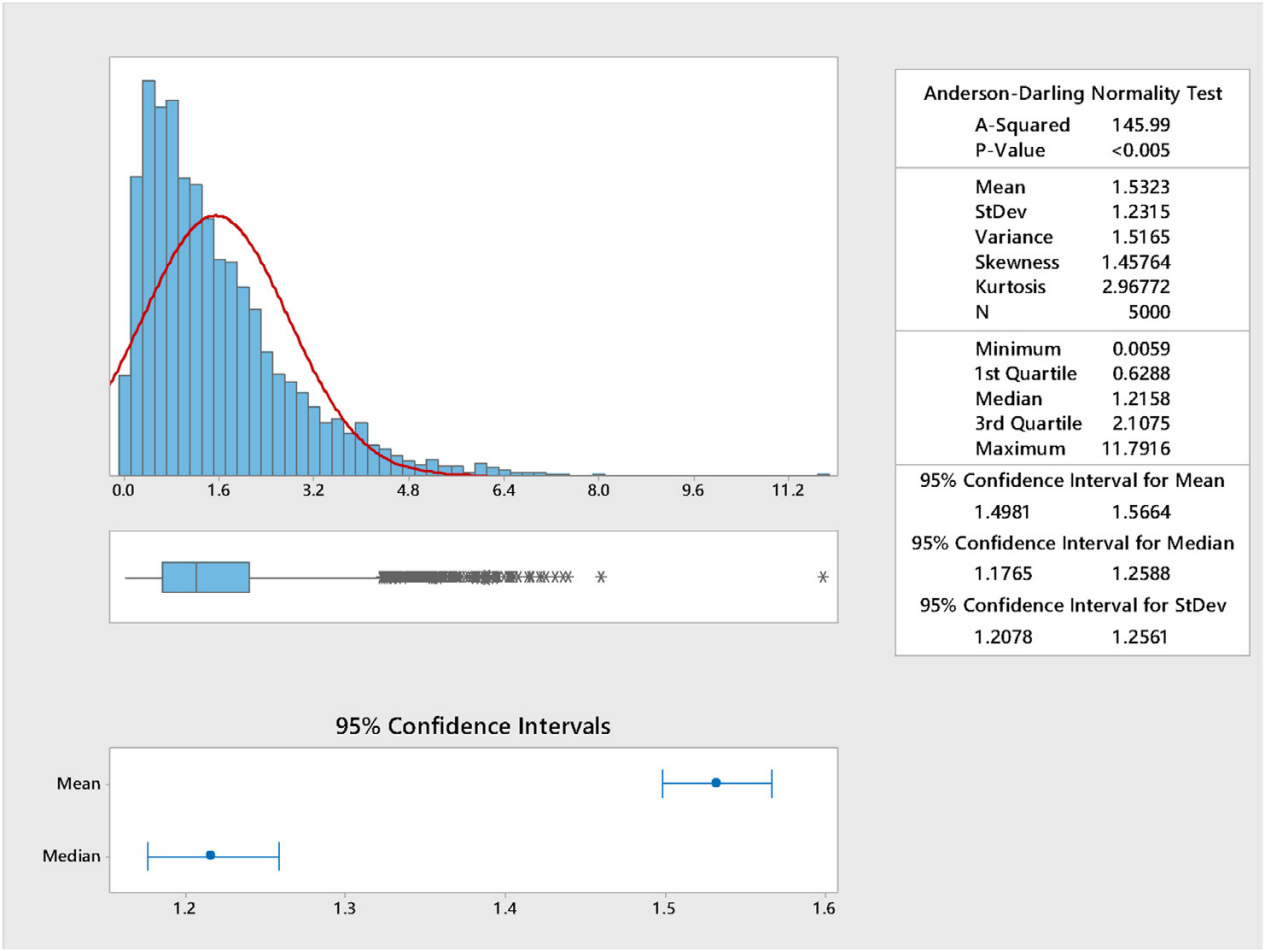
Graphical summary of the simulation for k = 1.5.

Simulation was done using Eq. (41) using p∈(0, 1) (p is from the continuous standard uniform distribution) and 5,000 sample size was used. The summary is presented in Figure 1.

From Figure 1, it could be observed that the simulation yielded values close to the theoretical values of quartiles and the comparison is presented in Table 13.

**Table 13:** Comparison of the statistical moments for k = 2.5

Statistic	R software	Approximate	Simulated
Mean	1.5	1.5	1.5323
Median	1.182986942	1.182986942	1.2158
Variance	1.5	1.5	1.5165
Skewness	1.63299	1.63299	1.45764
First quartile	0.606266452	0.606266452	0.6288
Third quartile	2.054172468	2.054172468	2.1075

#### 4.6.3 When k = 2.5

This is the particular case of Eq. (7) when k = 2.5 and λ=1. This implies that Eq. (7) reduces to;

(42) $$\frac{d^{2}Q}{dp^{2}}=\left[1-\frac{3}{2Q}\right]{\left(\frac{dQ}{dp}\right)}^{2}$$

The solution of Eq. (42) was obtained explicitly without the use of the initial values. The solution is presented as a complex closed form equation;

(43) $$c_{1}+p=\frac{\frac{3}{4}\sqrt{p}\;erf\left(\sqrt{Q(p)}\right)-\frac{1}{2}e^{-Q(p)}\sqrt{Q(p)}\left(2Q(p)+3\right)}{c_{2}}$$

(44) $$c_{1}c_{2}+c_{2}p=\frac{3}{4}\sqrt{p}\;erf\left(\sqrt{Q(p)}\right)-\frac{1}{2}e^{-Q(p)}\sqrt{Q(p)}\left(2Q(p)+3\right)$$

Fitting is done on Eq. (44) using the R software values of the quantile function. Hence, the right-hand-side of Eq. (44) $R(p)=\frac{3}{4}\sqrt{p}\;erf\left(\sqrt{Q(p)}\right)-\frac{1}{2}e^{-Q(p)}\sqrt{Q(p)}\left(2Q(p)+3\right)$ for different values of p are given in Table 14.

**Table 14:** The values of $R(p)=\frac{3}{4}\sqrt{p}\;erf\left(\sqrt{Q(p)}\right)-\frac{1}{2}e^{-Q(p)}\sqrt{Q(p)}\left(2Q(p)+3\right)$ for different quartiles.

p	R(p)	p	R(p)	p	R(p)
0.01	2.673527224	0.1	2.953772322	0.91	-1.560142904
0.02	2.899007467	0.2	2.451766414	0.92	-1.609883966
0.03	2.998470827	0.3	1.863038089	0.93	-1.659401181
0.04	3.044601377	0.4	1.261925998	0.94	-1.708695781
0.05	3.061240764	0.5	0.669179684	0.95	-1.757769461
0.06	3.059265593	0.6	0.092834283	0.96	-1.806624608
0.07	3.044560247	0.7	-0.463494181	0.97	-1.855264766
0.08	3.020666841	0.8	-0.998081196	0.98	-1.903695662
0.09	2.989880748	0.9	-1.510177087	0.99	-1.951928251

It can be seen from Table 14 that the values of R(p) decreases to zero for low values of p and approximates to -2 as p tends towards 1. Hence; $\lim_{R\left(p\right)\to 0}=0$ and $\lim_{R\left(p\right)\to 1}=-2$.

Initial values are assumed for $c_{2}=1$ and $c_{1}$ is obtained for different values of the quartiles that will make

(45) $$c_{1}+p=R(p)=\frac{3}{4}\sqrt{p}\;erf\left(\sqrt{Q(p)}\right)-\frac{1}{2}e^{-Q(p)}\sqrt{Q(p)}\left(2Q(p)+3\right)$$

The values of $c_{1}$ for different quartiles are presented in Table 15.

**Table 15:** Values of $c_{1}$ for different quartiles for k = 2.5

p	$c_{1}$	p	$c_{1}$	p	$c_{1}$
0.01	1.673527224	0.1	1.953772322	0.91	-2.560142904
0.02	1.899007467	0.2	1.451766414	0.92	-2.609883966
0.03	1.998470827	0.3	0.863038089	0.93	-2.659401181
0.04	2.044601377	0.4	0.261925998	0.94	-2.708695781
0.05	2.061240764	0.5	-0.330820316	0.95	-2.757769461
0.06	2.059265593	0.6	-0.907165717	0.96	-2.806624608
0.07	2.044560247	0.7	-1.463494181	0.97	-2.855264766
0.08	2.020666841	0.8	-1.998081196	0.98	-2.903695662
0.09	1.989880748	0.9	-2.510177087	0.99	-2.951928251

It can be seen from Table 15 that the values of $c_{1}$ decreases to -1 for low values of p and approximates to -3 as p tends towards 1. Hence; $\lim_{c_{1}\to 0}=-1$ and $\lim_{c_{1}\to 1}=-3$.

The complexity of Eq. (45) implies that Q(p) cannot be easily simplified. Hence simulation can be done using;

(46) Q(p)=B(p)R(p)

Where B(p) is computed using the R software values. The values of A(p) are presented in Table 16.

**Table 16:** Values of B(p) for different quartiles for k = 2.5

p	B(p)	p	B(p)	p	B(p)
0.01	0.103664192	0.1	0.272584988	0.91	-3.051346804
0.02	0.129680407	0.2	0.477723794	0.92	-3.055062195
0.03	0.150586089	0.3	0.805111863	0.93	-3.070694425
0.04	0.16936913	0.4	1.448381137	0.94	-3.100678358
0.05	0.187093456	0.5	3.251339136	0.95	-3.149018668
0.06	0.204283531	0.6	27.63993502	0.96	-3.222676088
0.07	0.221248172	0.7	-6.542077796	0.97	-3.335000672
0.08	0.238192481	0.8	-3.651644855	0.98	-3.516376821
0.09	0.25526574	0.9	-3.05803769	0.99	-3.864453641

It can be seen from Table 16 that the values of A(p) decreases to 0 for low values of p and approximates to -6 as p tends towards 1. Hence; $\lim_{A\left(p\right)\to 0}=Q{\left(p\right)}^{2}$ and $\lim_{A\left(p\right)\to 1}=-2Q\left(p\right)$ (This was the result as $p=10^{-56}$ and p≈1.

#### 4.6.4 Simulation

**Figure 2:**
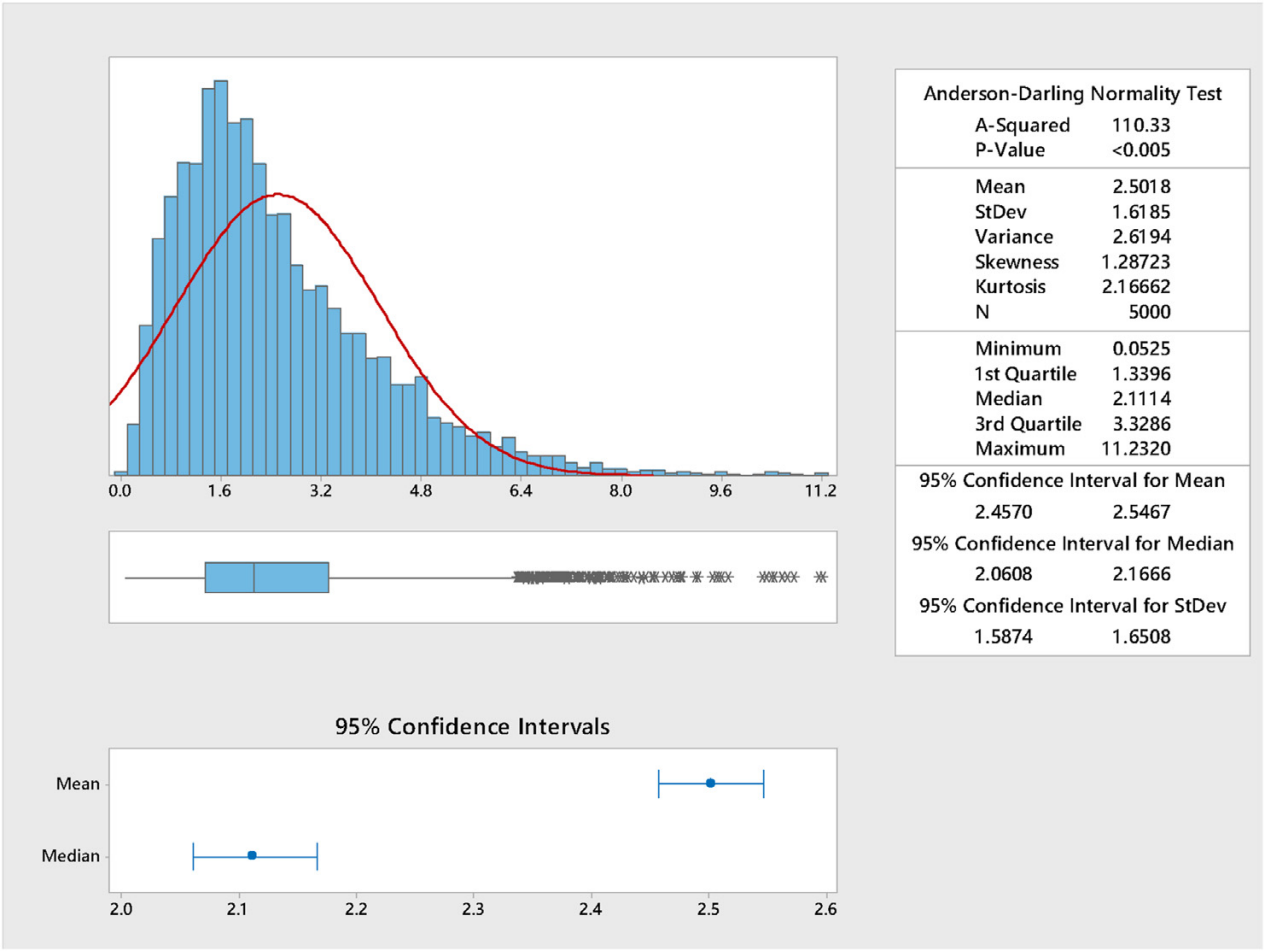
Graphical summary of the simulation for k = 2.5.

Simulation was done using Eq. (46) using p∈(0, 1) (p is from the continuous standard uniform distribution) and 5,000 sample size was used. The summary is presented in Figure 2.

From Figure 2, it could be observed that the simulation yielded values close to the theoretical or R software values of quartiles and the comparison is presented in Table 17.

**Table 17:** Comparison of the statistical moments for k = 2.5

Statistic	R software	Approximate	Simulated
Mean	2.5	2.5	2.5018
Median	2.175730096	2.175730096	2.1114
Variance	2.5	2.5	2.6194
Skewness	1.2649	1.2649	1.28723
First quartile	1.337301405	1.337301405	1.3396
Third quartile	3.312839882	3.312839882	3.3286

### 4.7 Error analysis

Error analysis was done to quantify the extent to which the results obtained from this work are close to the R software values. Absolute error and Kullback–Leibler divergence are used as performance metrics.

#### 4.7.1 Case 1

**Figure 3:**
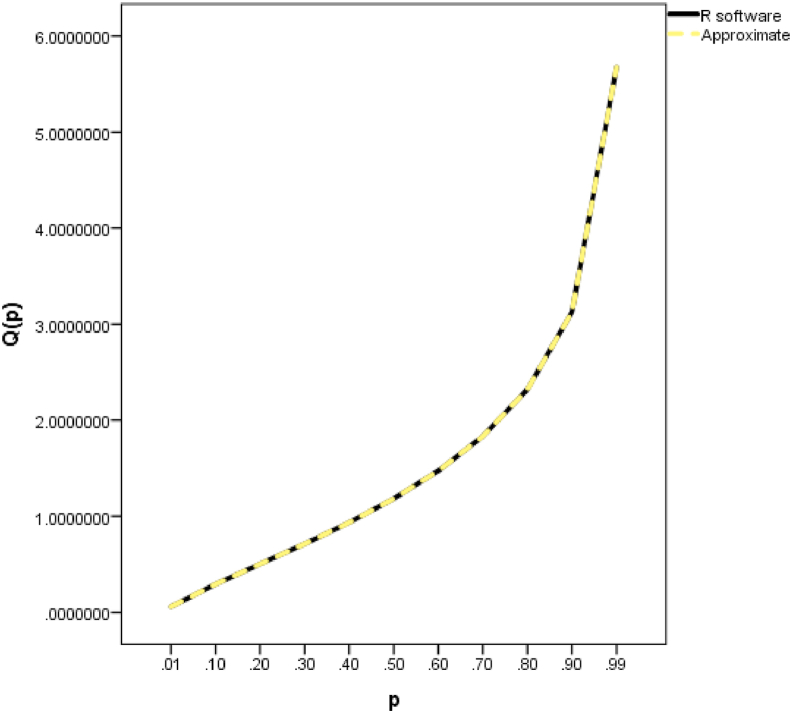
Line graph showing the R software and approximate values for k = 1.5.

The comparison using absolute error between the R software values and the approximate values obtained in this paper for the degrees of freedom equals 1.5 is presented in Table 18 and graphically in Figure 3.

**Table 18:** Absolute error analysis for k = 1.5

p	R software	This work	Error
0.01	0.057415901	0.057415901	0
0.02	0.09241591	0.09241591	0
0.03	0.122549354	0.122549354	0
0.04	0.150075709	0.150075709	2.77556E-17
0.05	0.175923159	0.175923159	2.77556E-17
0.06	0.200586679	0.200586679	2.77556E-17
0.07	0.224369351	0.224369351	0
0.08	0.247473781	0.247473781	0
0.09	0.270043996	0.270043996	0
0.1	0.292187187	0.292187187	0
0.2	0.502587007	0.502587007	0
0.3	0.711826122	0.711826122	0
0.4	0.934584202	0.934584202	0
0.5	1.182986942	1.182986942	0
0.6	1.473083037	1.473083037	0
0.7	1.832435392	1.832435392	2.22045E-16
0.8	2.320813838	2.320813838	0
0.9	3.125694316	3.125694316	4.44089E-16
0.91	3.245728858	3.245728858	4.44089E-16
0.92	3.37934631	3.37934631	4.44089E-16
0.93	3.530157084	3.530157084	0
0.94	3.703440022	3.703440022	0
0.95	3.907363952	3.907363952	4.44089E-16
0.96	4.155585455	4.155585455	0
0.97	4.473643749	4.473643749	0
0.98	4.918704656	4.918704656	0
0.99	5.672433365	5.672433365	0

A close look at Table 18 showed that the closed form expressions for the QF of the gamma distribution obtained from this paper are correct up to an average of 14 decimal points.

From Figure 3, the R software and the approximate ones are indistinguishable.

Kullback–Leibler divergence (D_KL_) was used to determine how this work performed with the R software values. This was done in two phases. In each phase, the R software values and the approximate values obtained from this work were used for p=0.01,0.02,0.03,...,0.99. Hence, $R_{i}:i=1,2,3,...,99$ are the R software quantile values while $A_{i}:i=1,2,3,...,99$ are the approximate values obtained in this work.

First, the D_KL_ of R software values given the approximate was obtained using the formula;

(47) $$D_{KL}\left(R\left|\;\right|A\right)=R_{1}\;\ln\frac{R_{1}}{A_{1}}+R_{2}\;\ln\frac{R_{2}}{A_{2}}+...+R_{99}\;\ln\frac{R_{99}}{A_{99}}$$

This was computed as $D_{KL}\left(R\left|\;\right|A\right)=2.78614\times 10^{-15}$

Second, the D_KL_ of the approximate given the R software values was obtained using the formula;

(48) $$D_{KL}\left(A\left|\;\right|\text{R}\right)=A_{1}\;\ln\frac{A_{1}}{R_{1}}+A_{2}\;\ln\frac{A_{2}}{R_{2}}+...+A_{99}\;\ln\frac{A_{99}}{R_{99}}$$

This was computed as $D_{KL}\left(A\left|\;\right|\text{R}\right)=2.33974\times 10^{-16}$

The two values of Kullback–Leibler divergence showed that the existence of small error between the R software and approximate values obtained in this paper.

#### 4.7.2 Case 2

**Figure 4:**
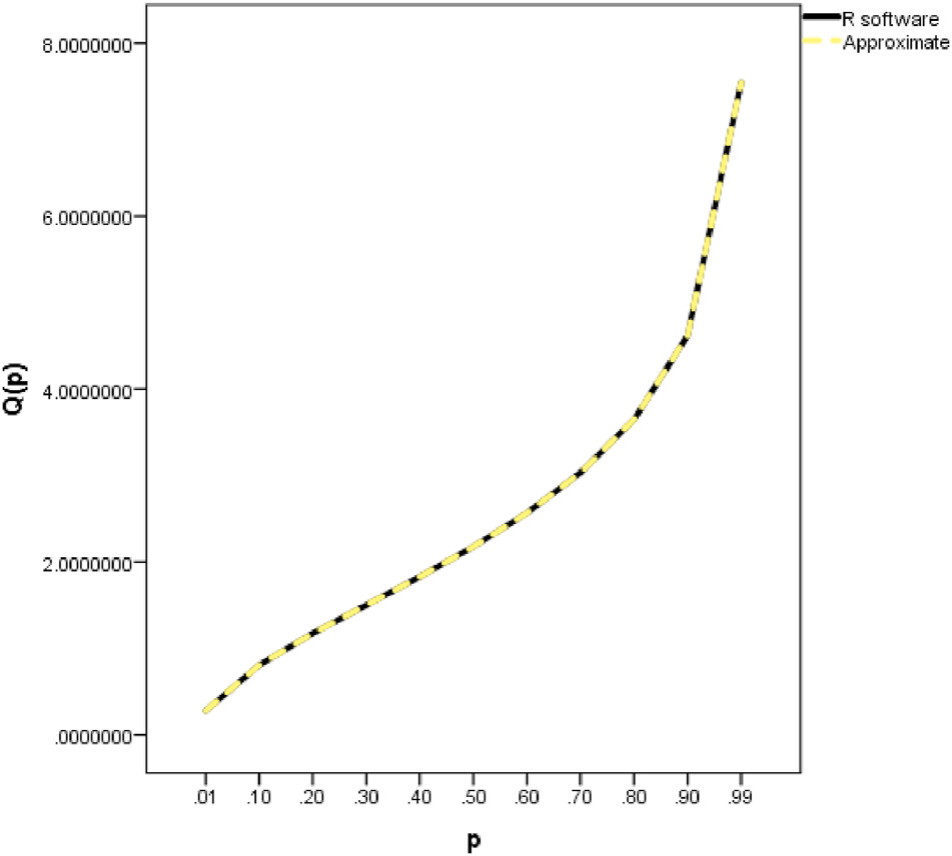
Line graph showing the R software and approximate values for k = 2.5.

The comparison using absolute error between the R software values and the approximate values obtained in this paper for the degrees of freedom equals 2.5 is presented in Table 19 and graphically in Figure 4.

**Table 19:** Absolute error analysis for k = 2.5

p	R software	This work	Error
0.01	0.277149038	0.277149038	0
0.02	0.375944467	0.375944467	5.55112E-17
0.03	0.451527995	0.451527995	0
0.04	0.515661485	0.515661485	0
0.05	0.572738113	0.572738113	0
0.06	0.624957578	0.624957578	0
0.07	0.67360339	0.67360339	0
0.08	0.719500128	0.719500128	0
0.09	0.76321412	0.76321412	0
0.1	0.805153993	0.805153993	0
0.2	1.171267153	1.171267153	0
0.3	1.499954066	1.499954066	0
0.4	1.827749812	1.827749812	2.22045E-16
0.5	2.175730096	2.175730096	0
0.6	2.565933537	2.565933537	0
0.7	3.032214992	3.032214992	0
0.8	3.644638063	3.644638063	0
0.9	4.61817845	4.61817845	0
0.91	4.760537065	4.760537065	0
0.92	4.918295642	4.918295642	0
0.93	5.095513954	5.095513954	0
0.94	5.298116031	5.298116031	0
0.95	5.535248847	5.535248847	0
0.96	5.822165924	5.822165924	0
0.97	6.18730924	6.18730924	0
0.98	6.6941113	6.6941113	0
0.99	7.543136235	7.543136235	0

A close look at Table 19 showed that the closed form expressions for the QF of the gamma distribution obtained from this paper are correct up to an average of 14 decimal points.

Similarly, in Figure 4, the R software and the approximate ones are indistinguishable.

Similarly; $D_{KL}\left(R\left|\;\right|A\right)=2.78614\times 10^{-15}$$D_{KL}\left(A\left|\;\right|\text{R}\right)=2.33974\times 10^{-16}$

The two values of Kullback–Leibler divergence showed that the existence of small error between the R software and approximate values obtained in this paper.

### 4.8 Limitation

The study is limited by the inability to obtain a single equation for both parameters of the gamma distribution. An attempt to get a single equation order ultimately reduces accuracy because any proposed curve will have difficulties with the extreme tails of the distribution.

## 5 Conclusion

The Quantile mechanics approach was used to obtain the closed-form expression for the quantile function of the gamma distribution for shape parameter (degrees of freedom) equals one to five. The case of one was not thoroughly analyzed because the closed-form is already existent in the literature. The ordinary differential equations obtained from quantile mechanics were solved explicitly and the product of quantile functions that are very close to the R software values, as shown in the error analysis. In addition, the quantile function obtained is the same as the R software values up to an average of 10 decimal points. The approach used in this paper can be used for any given degrees of freedom (shape parameter) of the gamma distribution. Secondly, the authors are more concern about precision and hence, the absolute error was used instead of root mean square to prove that the results from the present work are almost the same thing with the R software and in addition, unlike the R software, closed-form expressions obtained from the present work can be used in modeling and simulation. This work has provided evidence that the quantile function of the gamma distribution is a function of error and exponential functions. Unsurprisingly, exponential is linked to the exponential distribution whose sums yield the gamma distribution. This result will open up new areas on the use of gamma distribution in modeling fading channels, random number generation, distribution fit, and simulation of wireless systems described by the distribution. Moreover, algorithms and computer codes can be developed for implementation.

## Declarations

### Author contribution statement

Hilary Okagbue, Muminu O. Adamu & Timothy A. Anake: Conceived and designed the experiments; Performed the experiments; Analyzed and interpreted the data; Wrote the paper.

### Funding statement

This research did not receive any specific grant from funding agencies in the public, commercial, or not-for-profit sectors.

### Data availability statement

Data will be made available on request.

### Declaration of interests statement

The authors declare no conflict of interest.

### Additional information

No additional information is available for this paper.

## References

[1] Liu X. (2018). Secrecy analysis of a cloud-fog radio access network with binomial point process and general path-loss parameters. IEEE International Conference on Communications Workshops, ICC Workshops 2018.

[2] Pattanayak D.R., Rai S., Dwivedi V.K., Singh G. (2018). A statistical channel model for a decode-and-forward based dual hop mixed RF/FSO relay network. Opt. Quant. Electron..

[3] Alexandropoulos G.C., Peppas K.P. (2018). Secrecy outage analysis over correlated composite Nakagami- m/gamma fading channels. IEEE Commun. Lett..

[4] Tiwari K., Bharti B., Saini D.S. (2018). SER performance improvement in OFDM system over generalized K-fading channel. Adv. Intell. Syst. Comput..

[5] Das M., Sahu B., Bhanja U. (2017). Coverage analysis of mobile network in Nakagami fading channel. Wireless Pers. Commun..

[6] Büyükçorak S., Kurt G.K., Yongaçoǧluz A. (2017). An empirical study on gamma shadow fading based localization. 25th European Signal Processing Conference, EUSIPCO.

[7] Kishk M.A., Dhillon H.S. (2017). Effect of cell-selection on the effective fading distribution in a downlink K-tier HetNet. IEEE Wireless Commun. Lett..

[8] Cho K., Lee J., Kang C.G. (2017). Stochastic geometry-based coverage and rate analysis under Nakagami log-normal composite fading channel for downlink cellular networks. IEEE Commun. Lett..

[9] Hajji M., El Bouanani F. (2017). Performance analysis of mixed Weibull and Gamma-Gamma dual-hop RF/FSO transmission systems. International Conference on Wireless Networks and Mobile Communications, WINCOM 2017; Hotel Farah, Rabat, Rabat; Morocco.

[10] Mathur A., Bhatnagar M.R., Panigrahi B.K. (2016). Performance of a dual-hop wireless-powerline mixed cooperative system. 9th International Conference on Advanced Technologies for Communications, ATC 2016; Hanoi; Viet Nam.

[11] Olofsson T., Ahlén A., Gidlund M. (2016). Modeling of the fading Statistics of wireless sensor network channels in industrial environments. IEEE Trans. Signal Process..

[12] Lam H.Y., Luini L., Din J., Alhilali M.J., Jong S.L., Cuervo F. (2017). Impact of rain attenuation on 5G millimeter wave communication systems in equatorial Malaysia investigated through disdrometer data. 11th European Conference on Antennas and Propagation, EUCAP 2017; Paris; France.

[13] Csóka T., Polec J., Ilčiková I., Doboš J. (2016). Binary error models for wireless sensor networks. 23rd International Conference on Systems, Signals and Image Processing, IWSSIP 2016; Bratislava; Slovakia.

[14] Niu J., Li G.Y., Li Y., Fang D., Li X. (2017). Joint 3D beamforming and resource allocation for small cell wireless backhaul in HetNets. IEEE Commun. Lett..

[15] Miyagawa S., Yamada T., Ninagawa C. (2017). Generic and realistic spatial deployment modeling on data transmission performance evaluation of multi-hop smart grid metering system. IEEE Transact. Electr. Electron. Eng..

[16] Yasuda S., Yoshida H. (2018). Prediction of round trip delay for wireless networks by a two-state model. IEEE Wireless Communications and Networking Conference, WCNC 2018; Barcelona.

[17] Pana, C., Severi, S., De Abreu, G.T.F. Super-accurate source localization via multiple measurement vectors and compressed sensing techniques. In IEEE Wireless Communications and Networking Conference, WCNC 2018; Barcelona; 1-5.

[18] Elkotby H., Vu M. (2016). A probabilistic interference distribution model encompassing cellular LOS and NLOS mmWave propagation. IEEE Global Conference on Signal and Information Processing, GlobalSIP 2016; Washington; United States.

[19] Aminfar A., Ghobadi C., Amirani M.C. (2017). Diffusion adaptation through free space optical (FSO) wireless communication channels. 4th IEEE International Conference on Knowledge-Based Engineering and Innovation, KBEI 2017; Iran University of Science and Technology, Tehran.

[20] Sharma M., Chadha D., Chandra V. (2016). High-altitude platform for free-space optical communication: performance evaluation and reliability analysis. J. Opt. Commun. Netw..

[21] Madina E., El Maliani A.D., El Hassouni M., Alaoui S.O. (2016). Study of magnitude and extended relative phase information for color texture retrieval in L a b∗ color space. International Conference on Wireless Networks and Mobile Communications, WINCOM 2016; Hotel Medina PalaceFez; Morocco.

[22] Sumbiri, D., Afullo, T.J.O., Alonge, A.A. Modelling of rain drop size distribution for microwave and millimeter wave in central Africa. In Progress in Electromagnetics Research Symposium - Fall, PIERS - FALL 2017; Singapore; Singapore; Volume 2017, 2398-2404.

[23] Celebi H., Guvenc I. (2017). Load analysis and sleep mode optimization for energy-efficient 5G small cell networks. IEEE International Conference on Communications Workshops, ICC Workshops 2017; Paris; France.

[24] Yildiz D., Karagol S., Ozgonenel O. (2017). A hyperbolic location algorithm for various distributions of a Wireless Sensor Network. 5th International Istanbul Smart Grids and Cities Congress and Fair, ICSG 2017; Istanbul Congress Center, Istanbul; Turkey.

[25] Zhu C., Yu W. (2018). Stochastic modeling and analysis of user-centric network MIMO systems. IEEE Trans. Commun..

[26] Zhu C., Yu W. (2016). Stochastic analysis of user-centric network MIMO. 17th IEEE International Workshop on Signal Processing Advances in Wireless Communications, SPAWC 2016.

[27] Zhou S., Wang X., Cao N., Li X. (2018). Performance analysis of wireless powered communications with multiple antennas. IEEE Access.

[28] Rahman M.M., Misic J., Misic V.B. (2018). Formation of cognitive personal area networks (CPANs) using probabilistic rendezvous. IEEE Trans. Veh. Technol..

[29] Altinel D., Karabulut Kurt G. (2016). Energy harvesting from multiple RF sources in wireless fading channels. IEEE Trans. Veh. Technol..

[30] Tadrous J., Sabharwal A. (2016). Interactive app traffic: an action-based model and data-driven analysis. 14th International Symposium on Modeling and Optimization in Mobile, Ad Hoc, and Wireless Networks, WiOpt 2016; Tempe; United States.

[31] Sopin E., Gudkova I., Markova E., Ageyev K. (2016). Approximation of resource requirements distribution for the analysis of M2M traffic characteristics. CEUR Workshop Proc..

[32] Niu J., Li G.Y., Li Y., Fang D., Zheng J., Li X. (2017). Performance analysis on 3D beamforming for downlink in-band wireless backhaul for small cells. 86th IEEE Vehicular Technology Conference.

[33] El Tokhy M.S. (2018). Error analysis of wireless sensor network based on OFDM signal transmission algorithms for radiation detection. Ad Hoc Sens. Wirel. Netw..

[34] Ninos M.P., Nistazakis H.E., Latsas G.P., Tombras G.S., Konofaos N. (2017). PSK OFDM optical wireless communication systems with receiver's diversity over gamma-gamma turbulence channels and spatial jitter. 6th International Conference on Modern Circuits and Systems Technologies, MOCAST 2017; Aristotle University Research Dissemination Center (KEDEA)Thessaloniki; Greece.

[35] Zhang J., Li X., Ansari I.S., Liu Y., Qaraqe K.A. (2017). Performance analysis of dual-hop DF satellite relaying over κ-μ Shadowed fading channels. IEEE Wireless Communications and Networking Conference, WCNC 2017; San Francisco; United States.

[36] Devroye L. (1986). Nonuniform Random Variate Generation.

[37] Azzalini A. (1981). A note on the estimation of a distribution function and quantiles by a kernel method. Biometrika.

[38] Wilk M.B., Gnanadesikan R. (1968). Probability plotting methods for the analysis of data. Biometrika.

[39] Matsumoto M., Nishimura T. (1998). Mersenne Twister: a 623-dimensionally equidistributed uniform pseudo-random number generator. ACM Trans. Model Comput. Simulat.

[40] Steinbrecher G., Shaw W.T. (2008). Quantile mechanics. Eur. J. Appl. Math..

[41] Shaw W.T., Luu T., Brickman N. (2014). Quantile mechanics II: changes of variables in Monte Carlo methods and GPU-optimised normal quantiles. Eur. J. Appl. Math..

[42] Munir A.U.K. (2012). Series Representations and Approximation of Some Quantile Functions Appearing in Finance.

[43] Krishnamoorthy K., Mathew T., Mukherjee S. (2008). Normal-based methods for a gamma distribution: prediction and tolerance intervals and stress-strength reliability. Technometrics.

[44] DiCiccio T.J. (1987). Approximate inference for the generalized gamma distribution. Technometrics.

[45] Wallace D.L. (1958). Asymptotic approximations to distributions. Ann. Math. Stat..

[46] Thom H.C. (1968). Approximate convolution of the gamma and mixed gamma distributions. Mon. Weather Rev..

[47] Luu T. (2015). Efficient and accurate parallel inversion of the gamma distribution. SIAM J. Sci. Comput..

[48] DiDonato A.R., Morris A.H. (1986). Computation of the incomplete gamma function ratios and their inverse. ACM Trans. Math Software.

[49] Gil A., Segura J., Temme N.M. (2012). Efficient and accurate algorithms for the computation and inversion of the incomplete gamma function ratios. SIAM J. Sci. Comput..

[50] Marsaglia G., Tsang W.W. (2000). A simple method for generating gamma variables. ACM Trans. Math Software.

[51] Ulrich G., Watson L.T. (1987). A method for computer generation of variates from arbitrary continuous distributions. SIAM J. Sci. Stat. Comput..

[52] Hoshi K., Burges S.J. (1981). Approximate estimation of the derivative of a standard gamma quantile for use in confidence interval estimates. J. Hydrol..

[53] Best D.J., Roberts D.E. (1975). Algorithm AS 91: the percentage points of the χ2 distribution. J. Roy. Stat. Soc. Ser. C (Appl. Stat.).

[54] Okagbue H.I., Adamu M.O., Anake T.A. (2019). Closed form expressions for the quantile function of the Erlang distribution used in engineering models. Wireless Pers. Commun..

[55] Okagbue H.I., Adamu M.O., Anake T.A. (2019). Quantile mechanics: issues arising from critical review. Int. J. Adv. Appl. Sci..

[56] Okagbue H.I., Adamu M.O., Anake T.A. (2018). Ordinary differential equations of probability functions of convoluted distributions. Int. J. Adv. Appl. Sci..

[57] Okagbue H., Adamu M.O., Anake T.A. (2020). Closed form expression for the inverse cumulative distribution function of Nakagami distribution. Wireless Network.

[58] Okagbue H.I., Adamu M.O., Anake T.A. (2020). Closed-form expressions for the quantile function of the Chi square distribution using the hybrid of quantile mechanics and spline interpolation. Wireless Pers. Commun..

[59] Okagbue H.I., Adamu M.O., Anake T.A. (2019). Closed form expression of the quantile function of Maxwell-Boltzmann distribution. Adv. Appl. Stat..

